# Airway Response to Dual Bronchodilation and Its Association with Inflammatory Markers in Treatment-Naïve Chronic Obstructive Pulmonary Disease: A Quantitative Computed Tomography Study

**DOI:** 10.3390/life16091448

**Published:** 2026-08-31

**Authors:** Ieva Dimiene, Jurgita Zaveckiene, Kristina Bieksiene, Neringa Vaguliene, Deimante Hoppenot, Kestutis Malakauskas, Marius Zemaitis, Skaidrius Miliauskas

**Affiliations:** 1 Department of Pulmonology, Medical Academy, Lithuanian University of Health Sciences, 44307 Kaunas, Lithuania; kristina.bieksiene@lsmu.lt (K.B.); neringa.vaguliene@lsmu.lt (N.V.); deimante.hoppenot@lsmu.lt (D.H.); kestutis.malakauskas@lsmu.lt (K.M.); marius.zemaitis@lsmu.lt (M.Z.); skaidrius.miliauskas@lsmu.lt (S.M.); 2 Department of Radiology, Medical Academy, Lithuanian University of Health Sciences, 44307 Kaunas, Lithuania; jurgita.zaveckiene@lsmu.lt

**Keywords:** COPD, airway morphology, computed tomography, eosinophil, hs-CRP

## Abstract

**Background/Objectives**: Quantitative computed tomography (CT) can be used to characterize airway responses to therapies for chronic obstructive pulmonary disease (COPD), but prospective real-world data in treatment-naïve patients remain limited, and the role of inflammatory markers as potential predictors of structural airway response is poorly understood. **Methods**: We conducted a prospective real-world study in treatment-naïve patients with moderate-to-severe COPD (*n* = 47). Lung function and chest CT were assessed at baseline and after 12 weeks of tiotropium/olodaterol (*n* = 34). Blood eosinophil count and high-sensitivity C-reactive protein (hs-CRP) were measured at baseline. **Results**: Lung function improved after 12 weeks (*p* < 0.05). Airway lumen area normalized to body surface area (Ai/BSA) increased from 10.2 ± 2.6 to 12.2 ± 2.7 mm^2^/m^2^, whereas relative wall area (WA%) decreased from 58.3 (56.6–61.5) to 55.9 (54.4–57.9)% (both *p* < 0.001). Improvement in Ai/BSA did not differ between upper and lower lobes (*p* > 0.05). Higher baseline hs-CRP was associated with a smaller Ai/BSA increase (*r* = −0.454, *p* = 0.01). No statistically significant associations were detected between ΔAi/BSA and the evaluated baseline variables, including blood eosinophil count, Ai/BSA, age, smoking history and lung function measurements (all *p* > 0.05), or between blood eosinophil count and baseline Ai/BSA or WA% (both *p* > 0.05). **Conclusions**: Initial dual bronchodilator therapy improved lung function and CT-derived airway measurements. In this cohort, no statistically significant association was detected between blood eosinophil count and airway response, whereas higher hs-CRP was associated with a smaller Ai/BSA increase.

## 1. Introduction

Chronic obstructive pulmonary disease (COPD) is a complex lung condition characterized by persistent respiratory symptoms such as dyspnea, cough, and sputum production and/or exacerbations. It results from structural changes in the airways (e.g., bronchitis and bronchiolitis) and/or damage to the alveoli (emphysema), leading to ongoing and usually progressive airflow limitation [1,2].

Although spirometry remains the standard method for the diagnosis and follow-up of COPD, chest computed tomography (CT) provides complementary information by objectively quantifying airway and parenchymal abnormalities that are not captured by pulmonary function tests [1]. Quantitative CT enables detailed phenotypic characterization of obstructive airway diseases, including COPD and severe asthma, and has emerged as a valuable imaging biomarker for evaluating disease severity, structural remodeling, and response to treatment [1,3,4,5,6]. In most COPD cases, long-acting bronchodilators, including combinations of long-acting muscarinic antagonists (LAMAs) and long-acting β_2_-agonists (LABAs), are recommended as initial treatment [2]. LAMA/LABA combinations have been shown to reduce symptoms and exacerbation risk as well as improve lung function and quality of life [7]. Beyond their functional benefits, specific dual bronchodilator therapies have been shown to increase airway lumen area (Ai) and reduce airway wall dimensions, including relative wall area (WA%), as assessed by quantitative CT [8,9,10,11]. However, studies using quantitative chest CT to evaluate treatment-induced structural airway changes remain scarce, particularly prospective real-world studies with treatment-naïve patients. Studying treatment-naïve patients allows for the evaluation of the direct structural effects of bronchodilator therapy without the potential confounding influence of previously prescribed maintenance treatment.

A proportion of patients with COPD is characterized by eosinophilic airway inflammation, and blood eosinophil count has emerged as an important biomarker for predicting differential responses to inhaled therapies, particularly combinations with corticosteroids [12,13,14,15]. However, data regarding the association between blood eosinophil count and structural lung changes and lung function in COPD are conflicting. While some studies have reported better lung function and reduced bronchial wall thickness, others have demonstrated poorer lung function together with increased airway abnormalities, air trapping, and emphysema in patients with higher blood eosinophil counts, whereas others have found no association between blood eosinophil count and these structural or functional measures, indicating that the relationship between eosinophilic inflammation and COPD morphology remains an important area of research [16,17,18,19,20]. Despite the recognized role of eosinophilia in COPD, it remains unclear whether blood eosinophil count influences the anatomical response to bronchodilator therapy.

Furthermore, COPD is characterized by systemic inflammation, and high-sensitivity C-reactive protein (hs-CRP) has been proposed as a biomarker of disease severity, exacerbation risk and worse prognosis [21,22,23,24,25,26]. However, whether systemic inflammation has an impact on the structural airway response to inhaled therapy, as assessed by quantitative CT, remains uncertain.

Based on previous findings and the existing gaps in the literature, the primary objective of this study was to evaluate changes in airway luminal area normalized to body surface area (Ai/BSA) and WA% across different segmental bronchi, as well as changes in the arithmetic mean of these measurements, following 12 weeks of treatment with a fixed-dose (FD) combination of tiotropium/olodaterol (TIO/OLO) 5/5 µg once daily (OD) in treatment-naïve patients with COPD. The secondary objectives were to explore whether baseline blood eosinophil count, hs-CRP, Ai/BSA, age, smoking exposure, and pulmonary function parameters were associated with treatment-induced changes in airway morphology. In addition, we investigated the associations between baseline blood eosinophil count and CT-derived airway dimensions.

## 2. Materials and Methods

### 2.1. Study Design and Participants

**Figure 1:**
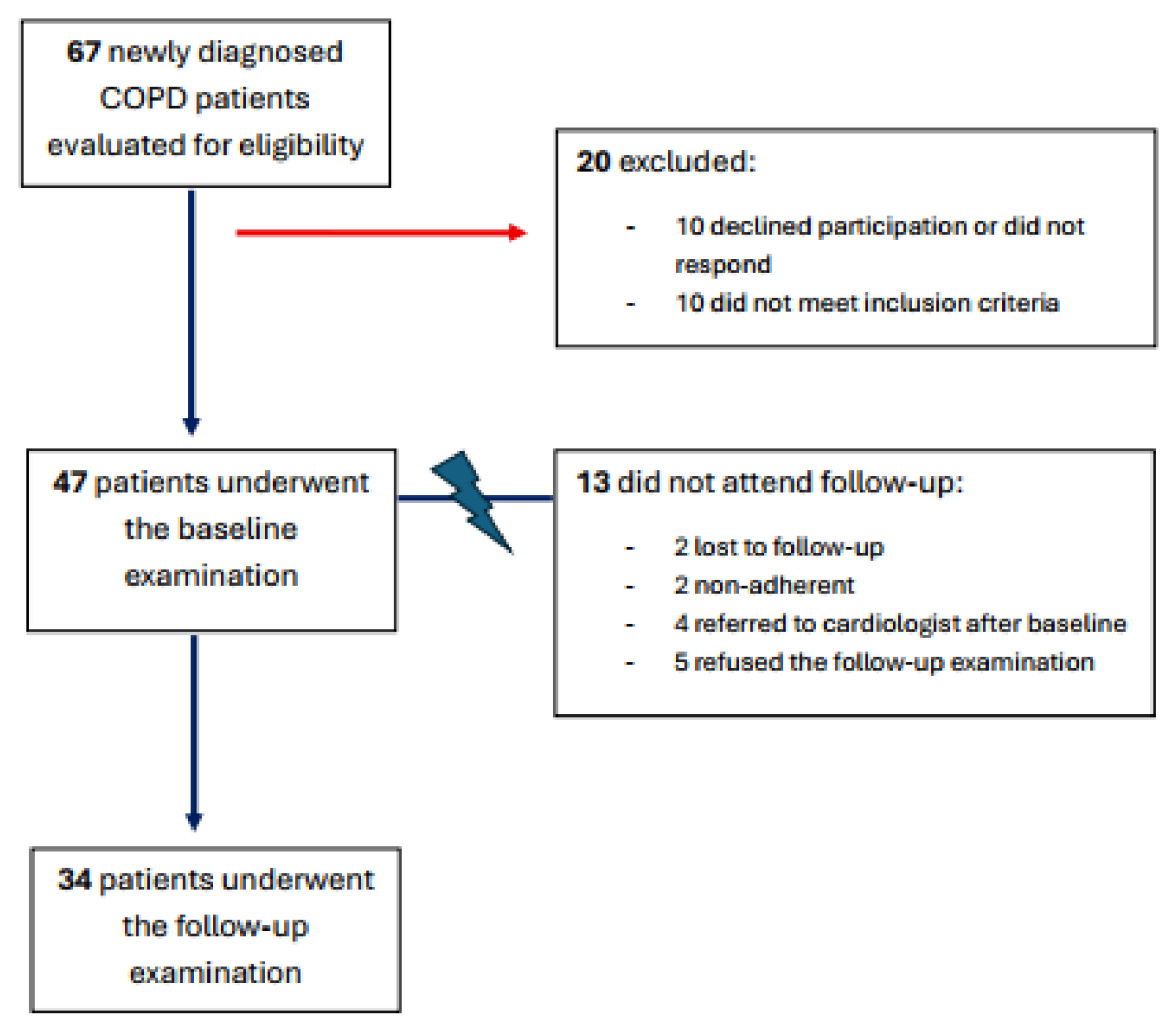
Participant selection and follow-up flow diagram.

This prospective, real-world observational study was conducted at the Hospital of Lithuanian University of Health Sciences Kauno Klinikos. Patients with newly diagnosed COPD were screened for eligibility during routine visits to the Department of Pulmonology between September 2021 and October 2025. No formal sample size or power calculation was conducted prior to the study. Instead, all eligible treatment-naïve patients with COPD who consented to participate were enrolled. A total of 47 treatment-naïve patients with newly diagnosed stable moderate-to-severe COPD were enrolled. Lung function, including spirometry, body plethysmography and gas diffusion, as well as full blood count and chest CT were performed and evaluated at baseline and after 12 weeks of treatment with TIO/OLO 5/5 µg FDC OD prescribed as a part of routine clinical care. Thirty-four patients attended both visits. Patients who did not complete the 12-week follow-up were either lost to follow-up, did not adhere to the prescribed treatment, were referred to a cardiologist after the baseline examination for additional cardiovascular evaluation and/or treatment adjustment, or refused to repeat examinations. The selection and follow-up of participants is presented in the flow diagram (Figure 1). The detailed inclusion and exclusion criteria were described previously [27,28]. The study protocol was approved by the Kaunas Regional Biomedical Research Ethics Committee (No. BE-2-46; 14 April 2021, Kaunas, Lithuania). This study was registered in the ClinicalTrials.gov database under identification number NCT06072690.

### 2.2. Lung Function Tests

The methods of spirometry, body plethysmography and gas diffusion have been described previously [27,28]. Lung function tests were conducted and interpreted according to the current European Respiratory Society and American Thoracic Society recommendations [29].

### 2.3. Blood Tests

Blood samples for full blood count and hs-CRP analysis were performed at baseline and processed in the Department of Laboratory Medicine of the Hospital of Lithuanian University of Health Sciences Kauno Klinikos according to established local laboratory protocols. For analyses involving blood eosinophil count, patients were categorized into two groups: <300 or ≥300 cells/µL. The reference values for hs-CRP were 0–1 mg/L. Due to technical issues, hs-CRP analyses were not performed for three patients; therefore, these patients were excluded from analyses with hs-CRP (all of the samples were in the group who completed both study visits).

### 2.4. Chest Computed Tomography Scan and Measurements of Segmental Bronchi

Chest CT examinations were performed using an Aquilion ONE TSX-301 CT scanner (Toshiba Medical Systems Corporation, Otawara, Japan), with image acquisition during end-inspiratory and end-expiratory phases. All subjects were instructed to hold their breath in the same way during the examination. CT examinations were performed during routine daytime visits 2 to 8 h after TIO/OLO administration. CT images were reconstructed using 1 mm thick slices. Segmental bronchi on inspiratory scans were evaluated using the Vitrea workstation (Canon Medical Informatics, Inc., Minnetonka, Minnesota, USA). We evaluated the standard segmental bronchi: right segmental bronchi 1 (RB1), 4 (RB4), and 10 (RB10), as well as left segmental bronchi 1 (LB1) and 10 (LB10) [30,31]. These bronchi were selected to provide standardized bilateral sampling of segmental airways from different lobar regions that could be consistently identified and measured. Ai (mm^2^) and WA% (%) were obtained using the semi-automated CT Respiratory Analysis software version 7.16 with manual correction performed when necessary. The measurements were performed by a pulmonologist and reviewed by an experienced radiologist. These measurements were obtained from the middle third of each airway. Segmental bronchi were evaluated separately and as the arithmetic mean of all five bronchi. Ai was normalized to body surface area (Ai/BSA) to account for individual differences in body measurements. The arithmetic mean of all measured segmental bronchi was used in the association analysis.

### 2.5. Statistical Methods

IBM SPSS Statistics for Windows, version 31.0.0.0 (IBM Corp., Armonk, NY, USA) was used for statistical analysis. Normality was assessed using the Shapiro–Wilk test. Differences between groups were assessed using the independent *t*-test for normally distributed variables and the Mann–Whitney test for variables that were non-normally distributed. The chi-square test or Fisher’s exact test was used for categorical variables. Within-patient changes from baseline to 12 weeks and differences in treatment-induced changes between upper and lower segmental bronchi were analyzed using the paired samples *t*-test for normally distributed variables and the Wilcoxon signed-rank test for non-normally distributed variables. Data are presented as mean ± standard deviation (SD) or median (interquartile range [IQR]), as appropriate. For correlation analysis we used Pearson’s correlation for normally distributed data and Spearman’s correlation for non-normally distributed data. Results were statistically significant when *p*-value was <0.05.

## 3. Results

### 3.1. Baseline Characteristics of Study Subjects

Out of 47 patients, 34 completed the follow-up evaluation. The baseline characteristics of completers and non-completers did not differ significantly (Table 1).

### 3.2. Changes in Lung Function Testing Results

A significant improvement in forced expiartory volume in 1 s (FEV_1_), residual volume (RV)-to-total lung capacity (TLC) ratio and functional residual capacity (FRC)-to-TLC were observed within 12 weeks of treatment (all *p* < 0.01). Gas diffusion did not change. The changes in lung function measurements are presented in Table 2.

### 3.3. Changes in Segmental Bronchi Measurements

The changes in the measurements of standard segmental bronchi (RB1, RB4, RB10, LB1 and LB10) were evaluated. Significant improvements in Ai/BSA of all measured bronchi were observed; accordingly, WA% decreased significantly in all bronchi except RB10. Detailed results of the changes in segmental bronchi after 12 weeks of treatment with TIO/OLO are presented in Table 3.

No statistically significant correlations were detected between ΔAi/BSA and changes in FEV_1_, RV/TLC, or FRC/TLC. The strongest association was observed for ΔFRC/TLC, although it did not reach statistical significance (*r* = −0.330, *p* = 0.057). The results are presented in Table 4.

When the median changes in Ai/BSA were compared between segmental bronchi of upper (RB1+LB1) and lower (RB10+LB10) lobes, no statistically significant difference was observed (2.0 [1.0–3.8] mm^2^ vs. 2.7 [0.4–3.5] mm^2^, respectively, *p* = 0.966).

### 3.4. Analysis of Associations Between Baseline Characteristics and Changes in Inner Airway Lumen

We found a negative moderate correlation between baseline hs-CRP and ΔAi/BSA. No other significant associations were detected between ΔAi/BSA and baseline variables, including Ai/BSA, age, smoking history, lung function measurements (FEV_1_, RV, or DLCOc), or blood eosinophil count ≥ or < 300 cells/µL (all *p* > 0.05). The results are presented in Table 5.

### 3.5. Analysis of Associations Between Baseline Blood Eosinophil Count and Baseline Airway Measurments

At baseline, blood eosinophil count (<300 vs. ≥300 cells/µL; *n* = 47) was not associated with CT-derived airway measurements. Mean Ai/BSA did not differ between patients with blood eosinophil counts <300 and ≥300 cells/µL (10.1 ± 2.7 vs. 10.4 ± 2.3 mm^2^/m^2^, mean difference: 0.3 mm^2^/m^2^, *p* = 0.715). Similarly, WA% was comparable between these groups (58.5 (IQR 57.9–61.1) vs. 58.1 (IQR 57.1–61.5)%, *p* = 0.552).

## 4. Discussion

In this prospective real-world study of newly diagnosed, treatment-naïve patients with COPD, 12 weeks of treatment with the TIO/OLO 5/5 µg FDC OD was associated with a mean relative increase of 22.2% in the arithmetic mean of Ai/BSA and a median relative reduction of 3.9% in the arithmetic mean of WA%, as assessed using quantitative chest CT. Although the magnitude of a relative Ai/BSA enlargement varied between individual segmental bronchi (16.1–28.9%), significant increases were observed in all evaluated bronchi. In addition, the improvement in Ai/BSA did not differ between the bronchi of upper and lower lobes (*p* > 0.05). We found that baseline hs-CRP value inversely correlated with the magnitude of ΔAi/BSA (*p* = 0.01). However, in this cohort, no statistically significant associations were detected between baseline blood eosinophil count or other baseline clinical characteristics and changes in airway morphology following treatment. In our cohort we did not observe any association between baseline blood eosinophil count and the measurements of baseline Ai/BSA and WA%.

The beneficial effects of dual bronchodilation on lung function are well established [32,33], and our spirometry and body plethysmography findings were consistent with previous reports. Nevertheless, to our knowledge, only a few studies have used quantitative chest CT to assess treatment-induced airway changes in patients with COPD. In 2014, Hoshino et al. performed a randomized, open-label, three-way clinical trial involving 54 patients with COPD. After 16 weeks of treatment with tiotropium/indacaterol (TIO/IND; *n* = 18), Ai/BSA improved by 1.4 mm^2^/m^2^ (IQR: 0.4–2.4; *p* < 0.001) and WA% was reduced by 4.7% (IQR: 2.5–6.0%; *p* < 0.001), compared to baseline. Therapy with TIO/IND caused greater improvements in airway dimensions than either tiotropium or indacaterol alone. They also found that the changes in WA% and Ai/BSA correlated with changes in FEV_1_ (*r* = −0.44, *p* < 0.01 and *r* = 0.37, *p* < 0.01, respectively) [8]. Another study by Hoshino et al. reported similar improvements in WA% and Ai/BSA in the TIO/IND group (*n* = 22), with greater changes than those in fluticasone/salmeterol group (*n* = 21) [11]. In 2016, Shimizu et al. reported that after 4–5 weeks of glycopyrronium/indacaterol (GLY/IND) treatment, segmental bronchial Ai increased by 25.69 ± 4.27% from baseline (*p* < 0.0001), with even greater improvements observed in the 4th to 6th generation bronchi. Moreover, they found significant moderate-to-strong correlations between ΔAi (%) and ΔFEV_1_% predicted from the 3rd to 6th bronchial generations after GLY/IND treatment (all *p* < 0.01) [10]. In our study, improvements in segmental airway dimensions were consistent with the findings reported by Hoshino et al. and Shimizu et al. However, different from these studies, we did not observe significant correlations between changes in lung function and changes in radiological airway measurements. This discrepancy in findings may be explained by differences in study population (including disease severity), treatment duration, CT acquisition and analysis methodology, and bronchial evaluation strategy. Hoshino et al. only evaluated RB1, whereas Shimizu et al. assessed airway changes across the third to sixth bronchial generations of all segments in the right lung. In contrast, our analysis included five segmental bronchi from different lobes of both lungs, and the arithmetic mean of these measurements was used to provide a more representative assessment of bilateral airway morphology and its changes. Although the comparative studies are not recent and the observed morphometric changes are compatible with known LAMA/LABA effects, our study adds relevant information by focusing explicitly on newly diagnosed, treatment-naïve patients with moderate-to-severe COPD.

Although higher blood eosinophil count has been shown to predict response to inhaled corticosteroid (ICS)-containing treatment in COPD [15,34], its relationship with CT-derived airway morphometric response to inhaled therapies has not been directly assessed. Because a higher blood eosinophil count may reflect eosinophilic airway inflammation and has been linked to structural lung abnormalities in patients with COPD [14,18,19,35], we evaluated whether baseline blood eosinophil count was associated with changes in Ai/BSA and WA% after 12 weeks of TIO/OLO therapy. However, no significant associations were found. This suggests that eosinophilic inflammation may not be a determinant of anatomical airway response to inhaled therapies, particularly in the absence of ICS-containing treatment. Nevertheless, our study had a relatively small sample size and no control group receiving ICS-containing regimens; therefore, the findings should be interpreted cautiously and considered exploratory.

Previous literature suggests that patients with COPD have increased inflammatory response, including higher CRP expression, in lung fibroblasts and epithelial cells [36]. Moreover, Ostridge et al. reported significant associations between CT-derived airway and lung tissue abnormalities and blood CRP concentration [37]. However, data regarding the association between systemic inflammation and CT-derived airway response after inhaled bronchodilator therapy remain limited. In this context, the inverse correlation between baseline hs-CRP and ΔAi/BSA observed in our study may suggest that a greater systemic inflammation is associated with a less favorable airway response to inhaled treatment, possibly due to inflammation-related airway remodeling. The observed association between hs-CRP and airway response may also have been influenced by COPD-related comorbidities or mucus plugging; however, these factors were not specifically assessed in our study.

In an exploratory analysis, the associations of baseline arithmetic mean of Ai/BSA, age, smoking exposure, and lung function measurements with ΔAi/BSA were evaluated; however, no statistically significant associations were detected in this cohort. Furthermore, we found no previous studies with which to compare these findings.

As mentioned previously, the data regarding the association between eosinophilia and airway and lung morphology remain conflicting. For example, Du et al. (*n* = 448) observed that COPD patients with higher blood eosinophil counts had less bronchial wall thickening on CT images [16]. Another study by Peng et al. (*n* = 728) found that patients with high blood and sputum eosinophil counts (*n* = 105) had greater levels of air trapping and emphysema than those with low blood and sputum eosinophil counts (*n* = 392; *p* < 0.01), as measured by the percentages of low-attenuation areas (LAAs) below −856 HU (LAA-856) at full expiration and below −950 HU (LAA-950) at full inspiration on chest CT [17]. Tan et al. (*n* = 1120) found that COPD patients with blood eosinophil counts ≥300 cells/µL had increased LAA-856 as well as increased estimated airway wall thickness for an idealized airway with a 10-mm internal perimeter (Pi10) compared with patients with lower eosinophil counts. Moreover, patients with elevated eosinophil counts had a reduction in the total number of CT-visible airways [18]. A smaller study by Nakamura et al. (*n* = 93) found significant associations between higher blood eosinophil counts and LAA-950 in patients with thin airway walls (*n* = 38), as well as between higher blood eosinophil counts and the wall area in patients with thick airway walls (*n* = 39) [19]. In contrast, Stival et al. (*n* = 110) did not find associations between higher blood eosinophil counts (*n* = 28) and CT-derived measurements—emphysema score and bronchial wall thickness—in patients with COPD [20]. Although the sample size of our study is relatively small and the findings should be interpreted cautiously, we believe our results showing no significant associations between blood eosinophil count and airway measurements at baseline in treatment-naïve COPD patients add value to the existing literature.

## 5. Strengths and Limitations of Our Study

A key strength of this study is that, to our knowledge, it is the first real-world study to evaluate CT-derived airway measurements after the initiation of dual bronchodilator therapy in treatment-naïve patients with COPD. Focusing on treatment-naïve patients is valuable because it limits the potential influence of previous therapies on the study outcomes.

Nevertheless, our study has limitations that need to be mentioned. First, due to the relatively small sample size, the statistical power may be limited; therefore, the findings should be interpreted with caution. Second, this was a single-group study, which did not allow us to compare treatment effects across different COPD therapies. Third, hs-CRP measurements were unavailable for three patients due to technical issues, reducing the sample size for analyses with hs-CRP, which might have limited the statistical power of these analyses. Additionally, quantitative airway assessment was limited to five segmental bronchi. Although these bronchi provided standardized bilateral sampling across different lobes, their measurements may not fully reflect the heterogenous and peripheral airway involvement of COPD or represent the entire bronchial tree. Finally, we did not assess LAAs, a CT-derived measure of emphysema and air trapping, or other potentially informative CT features, such as mucus plug scores, which may have added valuable information to our study. Future studies integrating airway morphometric, mucus plug, and parenchymal CT assessments may provide a more comprehensive characterization of structural treatment responses in COPD.

## 6. Conclusions

After 12 weeks of initial dual bronchodilator therapy, patients with newly diagnosed, treatment-naïve COPD showed significant improvements in lung function and CT-derived segmental airway dimensions, with similar responses in upper- and lower-lobe bronchi. These findings underscore the importance of promptly initiating appropriate maintenance therapy. In this cohort, no statistically significant associations were detected between baseline blood eosinophil count and either baseline airway dimensions or their changes over the treatment period. Higher baseline hs-CRP levels were associated with a smaller increase in Ai/BSA; however, the clinical and mechanistic significance of this finding remains uncertain. This exploratory finding requires further investigation in larger, controlled studies.

## Figures and Tables

**Table 1:** Baseline characteristics of completers (*n* = 34) and non-completers (*n* = 13).

Characteristic	Completers	Non-Completers	*p*-Value
Age, years	59.9 ± 8.2	64.5 ± 6.5	0.075
Gender, male:female	28:6	10:3	0.692
Smoking exposure, pack-years	37.5 (18.8–50.0)	27.0 (12.3–35.0)	0.120
BMI, kg/m^2^	27.3 ± 5.2	27.3 ± 3.5	0.979
FEV_1_, % pred.	60.6 ± 13.4	54.0 ± 13.4	0.137
RV, % pred.	155.5 (137.8–177.8)	135.0 (127.0–165.5)	0.207
DLCOc, % pred.	80.8 ± 21.0	73.1 ± 17.9	0.247
Eos ≥ 300 cells/µL, n (%)	12 (35.3)	4 (30.8)	1.000
hs-CRP, mg/L	2.2 (1.1–4.0)	2.7 (0.9–6.3)	0.653
Emphysema, n (%)	27 (79.4)	9 (69.2)	0.467

Abbreviations: BMI—body mass index; FEV_1_—forced expiratory volume in 1 s; pred.—predicted; RV—residual volume; DLCOc—diffusing capacity of the lungs adjusted for hemoglobin level, Eos—eosinophil count; hs-CRP—high-sensitivity C-reactive protein.

**Table 2:** Changes in lung function testing results after 12 weeks of TIO/OLO treatment.

Measurement	Baseline	12 Weeks	*p*-Value *
FEV_1_, % pred.	60.6 ± 13.4	68.3 ± 14.4	<0.001 *
RV, % pred.	155.5 (137.8–177.8)	144.0 (121.3–167.5)	0.059
RV/TLC, % pred.	132.5 (123.8–149.0)	123.0 (114.0–141.3)	0.002 *
FRC/TLC, % pred.	119.8 ± 15.1	113.6 ± 14.9	0.008 *
DLCOc, % pred.	80.8 ± 21.0	81.4 ± 20.7	0.748

Abbreviations: FEV_1_—forced expiratory volume in 1 s; pred.—predicted; RV—residual volume, TLC—total lung capacity; FRC—functional residual capacity; DLCOc—diffusing capacity of the lungs adjusted for hemoglobin level. * *p*-value < 0.05 indicates statistically significant results.

**Table 3:** Changes in segmental bronchial Ai/BSA and WA% after 12 weeks of TIO/OLO treatment.

Measurement	Baseline	12 Weeks	Relative Change, %	*p*-Value *
**RB1**				
Ai/BSA, mm^2^/m^2^	8.1 (6.2–11.2)	10.2 (7.9–13.5)	24.2 ± 29.2	<0.001
WA%, %	60.2 ± 5.9	56.6 ± 4.4	−5.5 (−10.6–(−1.5))	<0.001
**RB4**				
Ai/BSA, mm^2^/m^2^	6.7 (5.0–8.2)	7.5 (6.0– 9.7)	16.1 (2.0–31.3)	<0.001
WA%, %	62.1 (60.2–65.1)	59.7 (56.1–62.7)	−3.7 (−11.2–1.0)	0.002
**RB10**				
Ai/BSA, mm^2^/m^2^	9.0 (7.2–14.2)	12.5 (9.0–14.9)	22.0 ± 32.2	0.003
WA%, %	55.7 (53.5–61.0)	55.5 (54.0–58.0)	−1.6 (−10.0–2.1)	0.200
**LB1**				
Ai/BSA, mm^2^/m^2^	12.5 ± 3.8	15.3 ± 4.5	19.7 (3.7–44.7)	<0.001
WA%, %	56.6 (53.3–58.4)	54.3 (50.7–56.3)	−3.6 (−6.7–(−1.1))	<0.001
**LB10**				
Ai/BSA, mm^2^/m^2^	11.3 ± 4.3	14.3 ± 4.6	28.9 (4.1–48.0)	<0.001
WA%, %	58.8 (54.0–61.4)	53.9 (50.4–57.4)	−5.5 ± 8.8	<0.001
**Mean**				
Ai/BSA, mm^2^/m^2^	10.2 ± 2.6	12.2 ± 2.7	22.2 ± 17.3	<0.001
WA%, %	58.3 (56.6–61.5)	55.9 (54.4–57.9)	−3.9 (−7.6–(−1.4))	<0.001

Abbreviations: RB—right segmental bronchus; Ai/BSA—inner airway lumen normalized to body surface area; WA%—relative wall area; LB—left segmental bronchus. * *p*-value < 0.05 indicates statistically significant results.

**Table 4:** Correlations between changes in Ai/BSA and lung function parameters after 12 weeks of TIO/OLO treatment (*n* = 34).

Measurement	Analysis	Effect Size	*p*-Value *
ΔFEV_1_, %	Pearson correlation	r = 0.264	0.131
ΔRV/TLC, %	Pearson correlation	r = 0.065	0.717
ΔFRC/TLC, %	Pearson correlation	r = −0.330	0.057

Abbreviations: Δ (delta)—change, Ai/BSA—inner airway lumen normalized to body surface area; FEV_1_—forced expiratory volume in 1 s;; RV—residual volume; TLC—total lung capacity; FRC—functional residual capacity. * *p*-value < 0.05 indicates statistically significant results.

**Table 5:** Analysis of associations between baseline characteristics and mean ΔAi/BSA after 12 weeks of TIO/OLO treatment (*n* = 34).

Baseline	Analysis	Effect Size	*p*-Value *
Ai/BSA, mm^2^/m^2^	Pearson correlation	r = −0.282	0.106
Age, years	Pearson correlation	r = −0.184	0.297
Smoking exposure, pack years	Spearman correlation	r = −0.083	0.639
FEV_1_, % pred.	Pearson correlation	r = 0.123	0.488
FVC, % pred.	Pearson correlation	r = 0.063	0.723
RV, % pred.	Spearman correlation	r = −0.059	0.738
DLCOc, % pred.	Pearson correlation	r = −0.008	0.965
hs-CRP mg/L	Spearman correlation	r = −0.454	0.010 *
Eos ≥/< 300 cells/µL	Independent *t*-test	Mean difference = −0.270	0.630

Abbreviations: Ai/BSA—inner airway lumen normalized to body surface area; FEV_1_—forced expiratory volume in 1 s; pred.—predicted; FVC—forced vital capacity; RV—residual volume; DLCOc—diffusing capacity of the lungs adjusted for hemoglobin level; hs-CRP—high-sensitivity C-reactive protein; Eos—eosinophil count. * *p*-value < 0.05 indicates statistically significant results.

## Data Availability

The data supporting the conclusions of this article are available upon request from the corresponding author.
